# Future directions for immunotherapy in meningioma treatment

**DOI:** 10.18632/oncotarget.27994

**Published:** 2021-10-26

**Authors:** John W. Rutland, Jonathan T. Dullea, Raj K. Shrivastava

**Keywords:** meningioma, immunotherapy, molecular genetics

Meningiomas are the most common intracranial neoplasm accounting for roughly 37% of all intracranial tumors diagnosed in the United States [1]. These tumors display a wide range of clinical outcomes from favorable to dismal. The current standard of care for these lesions is maximal resection of tumor tissue in regions amenable to safe resection. Given that these tumors often abut vital neurovascular structures, gross total resection is not always possible. The extent of resection is one of the strongest predictors of recurrence [2]. Subtotal resection is associated with tumor regrowth that necessitates subsequent resection; Another important predictor of long-term oncologic outcomes is the World Health Organization (WHO) grade of the tumor. The WHO has defined three grades based on histopathological features that are used to guide prognostication and intervention [3]. Within these grades, there is substantial variability in oncologic outcomes. Generally, WHO grade I (benign) tumors have a low risk of recurrence; however, certain subsets of these tumors do recur [4]. With this in mind, there exists a need for further development of clinical biomarkers for disease progression to improve prognostication and eventual selection of appropriate post-resection treatment modalities. A greater understanding of the underlying tumor biology is required to refine the treatment paradigm for high-risk meningiomas. Patients with tumors that underwent sub-total resection or who have WHO high-grade neoplasms could benefit from this change in treatment.

Given that meningiomas reside outside of the blood-brain barrier, they are susceptible to systemic immune modulation [5]. A promising area of current research is the relationship between lymphocyte infiltration and meningioma genetic characteristics and oncological outcomes. Understanding these immune-genetic interactions could help elucidate possible immunological therapy mechanisms, which may be effective in treating recurrent or high-risk of recurrence meningiomas. A recent study detailing the relationship between inflammatory cell infiltration and the genetic profile of meningiomas has provided preliminary insight into this area of research [6]. In this study, a relationship between scattered lymphocyte pattern and mutational burden was demonstrated, showing that meningiomas with scattered lymphocytes had an average mutational burden of 6.9 mutations, compared with tumors without scattered lymphocytes which had an average of 2.3 mutations [6]. This indicates that scattered lymphocytes are a predictor of high mutational burden. This study also demonstrated that NF2 mutations were associated with an increased infiltration of scattered lymphocytes. Previous studies in other neoplasms have demonstrated a relationship between other specific mutations, such as POLE, lymphocyte infiltration, and survival; similar relationships remain to be further investigated in meningiomas [7].

Our prior work suggests that a distinct subset of meningiomas characterized by higher mutational burden elicits a specific immune response, opening the possibility that well-selected meningiomas may respond favorably to immunological therapy. Prior studies in lung cancer and melanoma have demonstrated a stronger response to immunological treatment in high mutational burden sub-types [8]. The increased expression of tumor neoantigens resulting from the greater number of mutations likely explains the increase in the immune response to highly mutated tumors. The higher antigenic load results in a greater potential immune response both before and upon immunotherapy administration. Thus, the presence of lymphocytes may serve as an important biomarker for the presence of a high mutational burden, and infiltrating lymphocyte patterns may be useful in identifying certain tumors as amenable to treatment with immunological therapy.

While these studies have demonstrated preliminary data, future work is needed to increase our understanding of the complex relationships between immune environment and molecular genetic features of meningiomas. Specifically, the relationship between certain lymphocyte sub-classes and individual tumor mutation is required. A significant limitation of the current body of work is the lack of information on the class (CD4+ vs. CD8+) of lymphocytes present in the resected sample. Quantitative characterization of the sub-type would allow correlation of genetics with infiltration of specific genetic alterations. Identifying specific mutations associated with increased infiltration of specific immune cells could help to elucidate the infiltration's underlying mechanisms. This future work could also examine the effects of these identified mutations on critical oncologic endpoints such as recurrence and overall survival. Additionally, a focus on immune cell distribution and their special location within a tumor, which has been performed in the setting of other aggressive neoplasms, would be of great interest in the field of meningioma research [9]. These investigations will help to lay the theoretical basis for a mechanistic understanding of future meningioma immune therapy.

In envisioning the future of treating meningiomas, we anticipate a management algorithm by which a patient first undergoes a maximal safe resection with subsequent molecular genetic genotyping of resected tumor. Based on the genetic and histologic characteristics, including immune cell infiltration, the patient would then be directed towards an appropriate treatment plan. Histopathologic evaluation would include the characterization of lymphocytic infiltration and genetic characteristics. For a subset of patients identified as low risk of recurrence—by genetic, histologic, and resection extent characteristics—follow-up would consist of only interval surveillance MRI. For patients determined to be high-risk by genetics and histological features, treatment may involve adjuvant radiation therapy. The treatment arms mentioned above are not novel. We envision the novel addition of adjuvant immunotherapy for patients for whom gross total resection was not possible and who have tumor biology amenable to immunological treatment, based on quantitative analysis of immune cell phenotype. These markers could include the presence of scattered lymphocytes indicating a high mutational burden.
